# Methylation of CpG sites in RNF180 DNA promoter prediction poor survival of gastric cancer

**DOI:** 10.18632/oncotarget.1888

**Published:** 2014-04-08

**Authors:** Jingyu Deng, Han Liang, Guoguang Ying, Rupeng Zhang, Baogui Wang, Jun Yu, Daiming Fan, Xishan Hao

**Affiliations:** ^1^ Department of Gastroenterology, Tianjin Medical University Cancer Hospital, City Key Laboratory of Tianjin Cancer Center and National Clinical Research Center for Cancer, Tianjin, China; ^2^ Central laboratory, Tianjin Medical University Cancer Hospital, City Key Laboratory of Tianjin Cancer Center and National Clinical Research Center for Cancer, Tianjin, China; ^3^ Institute of Digestive Disease, Li Ka Shing Institute of Health Science, Chinese University of HongKong, Shatin, HongKong; ^4^ State Key Laboratory of Cancer Biology and Institute of Digestive Diseases, Xijing Hospital, Fourth Military Medical University, Xi'an, China

**Keywords:** Ring finger protein 180, Lymph node, Methylation, Prognosis, Gastric cancer

## Abstract

RNF 180, a novel tumor suppressor, has been implicated in the carcinogenesis and progress of gastric cancer. At present study, we found that lower expression of RNA180 was specific in gastric cancer tissues, and the inconsistently methylated levels of RNF180 promoter were identified in the gastric cancer tissues. Importantly, we demonstrated that the methylated CpG site count and four hypermethylated CpG sites (−116, −80, +97, and +102) were significantly associated with the survival of 400 gastric cancer patients, respectively. With multivariate survival analyses, we demonstrated that both the methylation of combined CpG (−116, −80, +97, and +102) sites and N stage were the independent indictor of prognosis of gastric cancer patients. Eventually, the methylation of combined CpG (-116, -80, +97, and +102) sites was identified to have smaller AIC value than N stage by mean of AIC calculation with the Cox proportional hazards model. These findings indicated that the quantitative detection of RNF180 promoter methylation had the intensive feasibility for evaluation the prognosis of gastric cancer patients in clinic.

## INTRODUCTION

Gastric cancer is the most common gastric malignant tumor and the second leading cause of death due to cancer worldwide [1, 2]. Although it is a consensus that depth of tumor invasion and number of lymph node metastasis are most intensive factors to evaluate the progression and prognosis of gastric cancer, researchers demonstrated that tumor-node-metastasis (TNM) classification was imprecise for prediction the survival of patients even in the latest version [3]. Therefore, biomarker detection is considered as a promising method to enhance the accurately prognostic prediction of gastric cancer. As we know, DNA methylation in the promoter region of a tumor suppressor gene can lead to transcriptional inactivation, which may be associated with the carcinogenesis of gastric cancer [4]. Ring finger protein 180 (RNF180), a novel member of the RING finger protein family and functions as an E3 ubiquitin ligase, was reported to participate in many biological processes including tumorigenesis [5]. Researchers demonstrated that promoter methylation of RNF180 DNA was more frequently detected in the gastric cancer tissue samples, which led to low or loss RNF180 expression in gastric cancer patients with poor overall survival (OS) [6]. The aim of this study was to detect the methylation of CpG sites of RNF180 promoter in the large scale patients with gastric cancer in order to determine which methylated CpG site of RNF180 promoter can convey worse prognosis in the China.

## RESULTS

### Patient Demographics and the Methylation of RNF180 Promoter

All 400 gastric cancer patients' clinicopathological characteristics are listed in Table 1. The 5-year survival rate (5-YSR) of all gastric cancer patients was 11.8%. The median OS of all patients was 21 months and 52 patients was alive when fellow-up was over.

**Table 1:** Patient Demographics and Survival Analysis of the 400 Patient Cohort

Characteristics	Case	Median OS (months)	x2 Value	Univariate P Value	Hazard Ratio (95% CI)	MultivariateP Value	AIC Value
Gender			0.919	0.338			
Male	274	22					
Female	126	20					
Age at surgery			0.203	0.652			
≤ 60	239	20					
> 60	161	23					
Tumor location			6.326	0.097			
Lower third	173	25					
Middle third	104	18					
Upper third	101	21					
> 2/3 stomach	22	18					
Tumor size			1.332	0.248			
< 4.0 cm	58	25					
≥ 4.0 cm	342	21					
T stage			10.980	0.012	1.109 (0.887−1.388)	0.363	
T1	4	55					
T2	17	19					
T3	26	33					
T4	353	20					
N stage			74.138	< 0.001	1.452 (1.249−1.688)	< 0.001	72.070
N0	98	35					
N1	59	27					
N2	87	21					
N3	159	14					
Location of lymph node metastasis tage)			41.221	< 0.001	0.958 (0.767−1.195)	0.702	
No	98	35					
Perigastric	132	20					
Extragastric	170	17					
Lauren classification			8.516	< 0.014	1.178 (0.952−1.458)	0.131	
Intestinal	112	26					
Diffuse	273	20					
Mixed	15	17					
Methylated CpG site count of RNF180 promoter			9.651	0.002	0.755 (0.514−1.110)	0.153	
Seven or less	195	24					
Eight or more	205	18					
Methylated status of CpG −116			4.165	0.041	1.329 (0.908−1.946)	0.144	
U	230	23					
M	170	19					
Methylated status of CpG −80			4.003	0.045	1.029 (0.741−1.430)	0.864	
U	272	23					
M	128	19					
Methylated status of CpG +97			5.328	0.021	1.268 (0.844−1.903)	0.253	
U	239	23					
M	161	19					
Methylated status of CpG +102			4.368	0.037	0.972 (0.767−1.195)	0.874	
U	252	23					
M	148	18					
Methylated status of combination CpG (−116, −80, +97, and +102) sites			12.568	< 0.001	1.752 (1.147−2.677)	0.010	57.586
U	186	24					
M	214	19					

Abbreviations: U, unmethylation; M, methylation.

### Immunohistochemical Staining for RNF180 protein in Gastric Cancer and Paired Adjacent Non-tumor Tissues

**Fig. 1:**
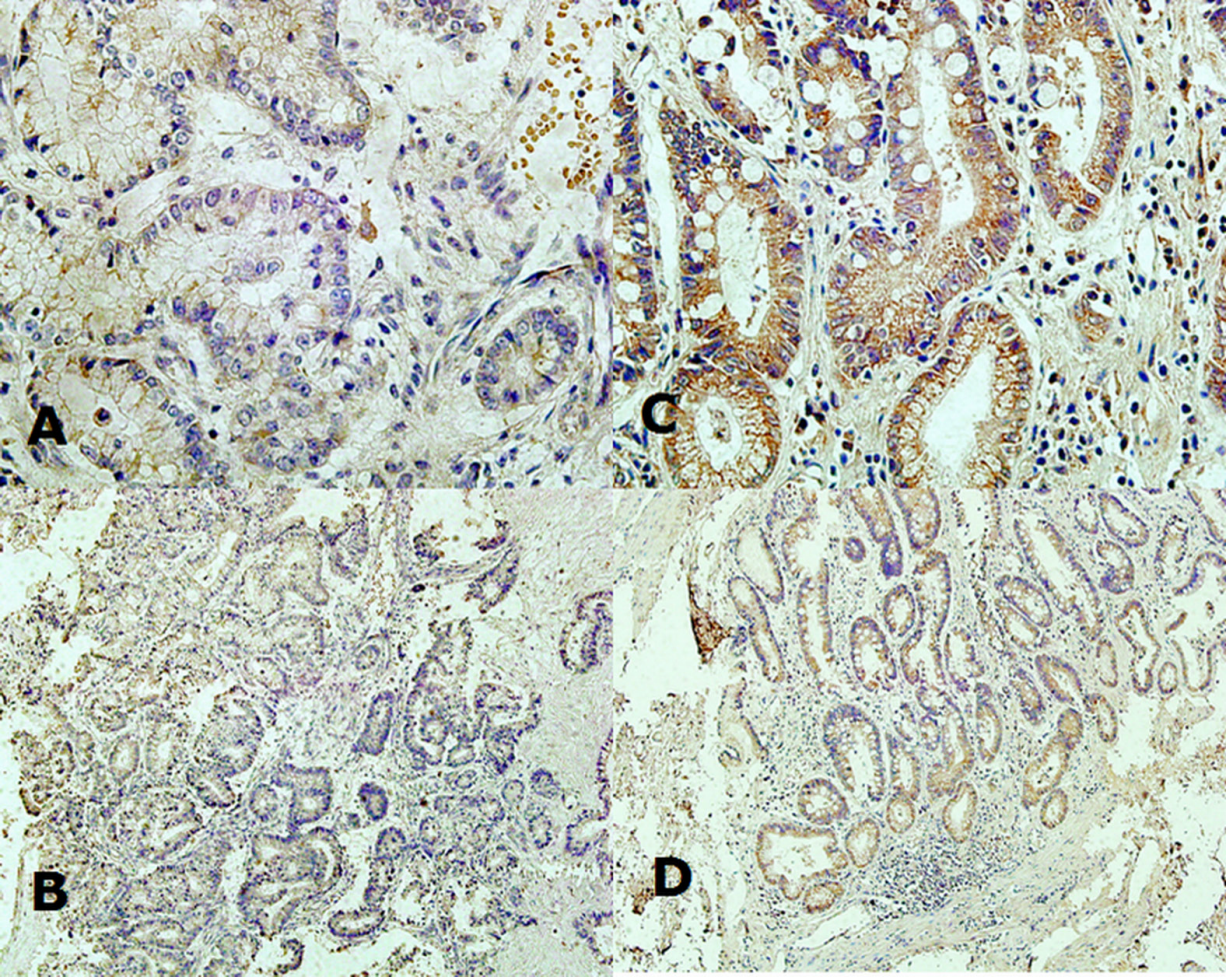
(A) Expression of RNF180 protein in the cytoplasm of malignant epithelium of gastric cancer tissue (x400 magnification); (B) Expression of RNF180 protein in the cytoplasm of malignant epithelium of gastric cancer tissue (x100 magnification); (C) Expression of RNF180 in the cytoplasm of epithelium of paired adjacent non-tumor tissue (x400 magnification); (D) Expression of RNF180 in the cytoplasm of epithelium of paired adjacent non-tumor tissue (x100 magnification).

RNF180 protein expression was mainly observed in the cytoplasm (Figure 1). RNF180 protein expression was detected in 38 (56.72%) (−), 13 (19.40%) (+), 10 (14.92%) (++), and 6 (8.96%) (+++) tumor samples, which represented that only 16 (23.88%) patients presented positive RNF180 protein expression. Meanwhile, we found that 7 (10.45%) (−), 21 (31.34%) (+), 19 (28.36%) (++), and 20 (29.85%) (+++) of RNF180 protein expression were detected in paired adjacent non-tumor tissues, respectively. Therefore, we demonstrated that the positive rate of RNF180 protein expression in gastric cancer tissues was significantly lower than that in adjacent non-tumor tissues (*P* <0.001).

### Western Blot Analysis for RNF180 Protein Expression in Gastric Cancer and Adjacent Non-tumor Tissues

**Fig. 2:**
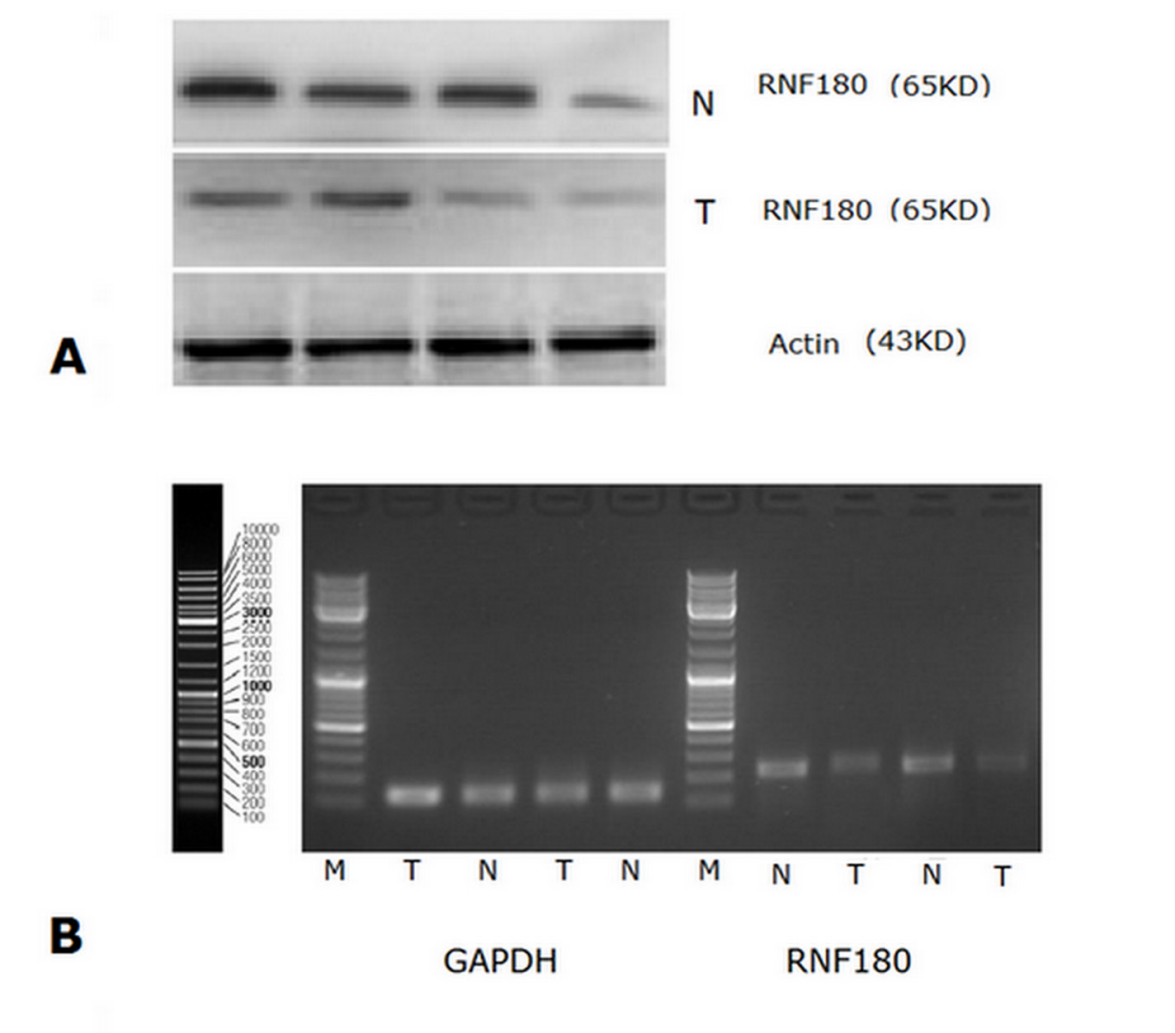
(A) Western Blot analysis for RNF180 protein expression in gastric cancer tissues and in normal gastric mucosal tissues; (B) RNF180 mRNA expression (RT-PCR) in gastric cancer tissues and in normal gastric mucosal tissues. (Representation: T, gastric cancer tissues; N, normal gastric mucosal tissues)

RNF180 protein expression was also detected in 67 gastric cancer tissues and 67 paired adjacent non-tumor tissues by Western blot, simultaneously (Figure 2A). The relative protein expression values of RNF180 in gastric cancer tissues was significantly lower than those in paired adjacent non-tumor tissues (0.454±0.054 VS 1.618±0.525, *P* =0.024). RNF180 protein expression in paired adjacent non-tumor tissues was about 3.56-fold higher than that in gastric cancer tissues.

### Expression of RNF180 mRNA in Gastric Cancer and Paired Adjacent Non-tumor Tissues

RNF180 mRNA expression was detected in 67 gastric cancer tissues and 67 paired adjacent non-tumor tissues (Figure 2B). The relative mRNA expression value of RNF180 in gastric cancer tissues was significantly lower than that in paired adjacent non-tumor tissues (0.632±0.285 VS 2.270±0.421, *P* <0.001). RNF180 mRNA expression in paired adjacent non-tumor tissues was about 3.60-fold higher than that in gastric cancer tissues.

### Methylation Detection of RNF180 Promoter

**Fig. 3:**
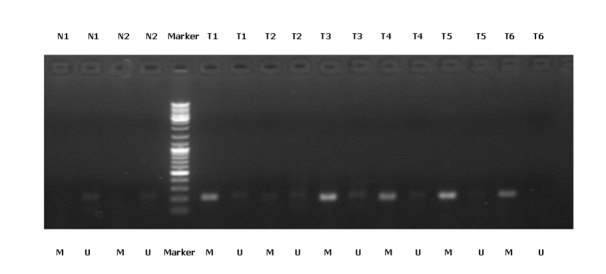
MSP detection of RNF180 promoter methylation in different gastric cancer tissues and normal gastric mucosal tissues. (Representation: T, gastric cancer tissues; N, normal gastric mucosal tissues; M, methylated; U, unmethylated)

We detected the qualitative degrees of RNF180 promoter methylation in 67 gastric cancer tissues with the MSP analysis (including 12 cases with methylation, 33 cases with partial methylation, and 22 cases without non-methylation), while no RNF180 promoter methylation was found in 25 normal gastric mucosal tissues (Figure 3).

**Fig. 4:**
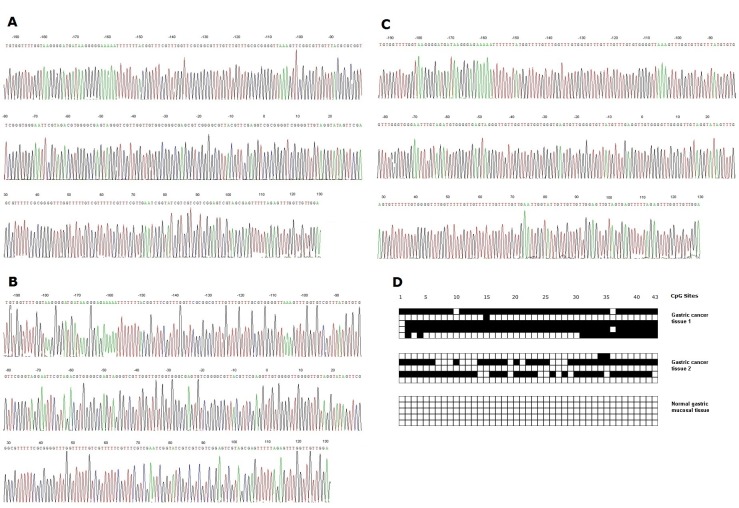
(A) Bisulphite sequencing figure of gastric cancer tissue 1, (B) Bisulphite sequencing figure of gastric cancer tissue 2, (C) Bisulphite sequencing figure of normal gastric mucosal tissue, and (D) Bisulfite sequencing results in gastric cancer tissues and in normal gastric mucosal tissue.

Subsequently, we adopted the quantitatively methylated analysis in all 400 gastric cancer samples by using the BGS method. Of these gastric cancer patients included in the study, 341 patients (85.25%) presented with one or more methylated CpG sites and 59 patients (14.75%) presented with no methylated CpG site. Methylated CpG site count of patients ranged between 0 and 43, with an average methylated CpG site count of 16.45. According to the result of cut-point analysis for the methylated CpG site count, 205 patients (51.25%) presented with eight or more methylated CpG sites and 195 patients (48.75%) presented with seven or less methylated CpG sites. No methylated CpG site was found in the normal gastric mucosal epithelial tissues. The methylation sequencing chart and CpG site chart were shown in Figure 4.

### Survival Analysis

**Fig. 5:**
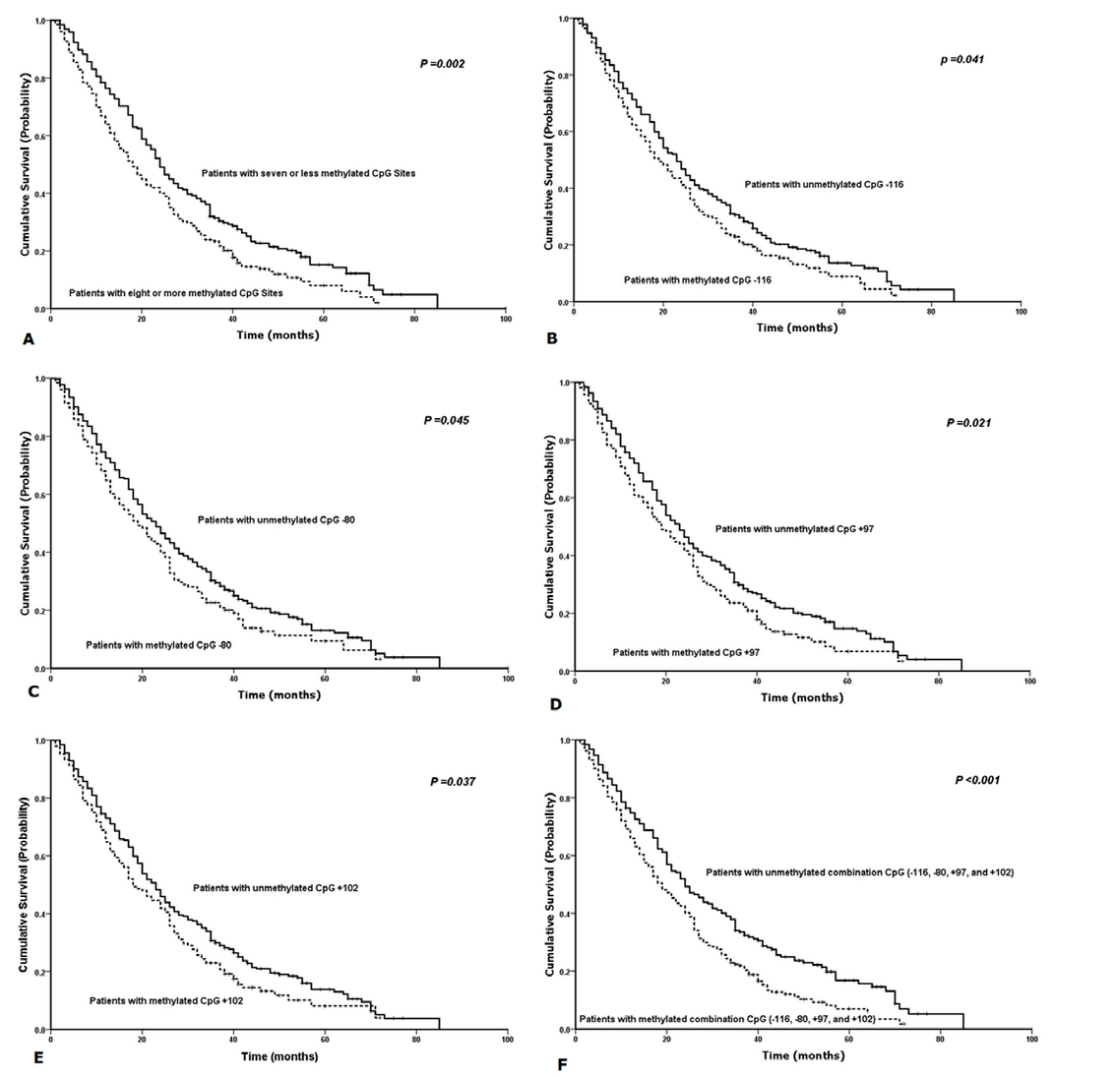
Kaplan-Meier survival curves comparing months of survival in gastric cancer patients are shown for (A) methylated CpG site count of RNF180 promoter, (B) methylated status of CpG −116, (C) methylated status of CpG −80, (D) methylated status of CpG +97, (E) methylated status of CpG +102, and (F) methylation of combined CpG (−116, −80, +97, and +102) sites.

With the univariate survival analysis, four clinicopathological characteristics were found to have statistically significant associations with OS of gastric cancer patients. They were as follows: T stage (P = 0.012), N stage (P < 0.001), extent of lymph node metastasis (P < 0.001), and Lauren classification (P = 0.014) (Table 1). Beside, we also demonstrated that the methylation of CpG −116 (P = 0.041), the methylation of CpG −80 (P = 0.045), the methylation of CpG +97 (P = 0.021), the methylation of CpG +102 (P = 0.037), the methylated CpG site count (P = 0.002), and the methylation of combined CpG (−116, −80, +97, and +102) sites (P < 0.001) were significantly associated with the OS of patients with the Kaplain-Meier curves discrimination (Table 1) (Figure 5). Methylation of combined CpG (−116, −80, +97, and +102) sites indicates that anyone of 4 CpG sites (CpG −116, CpG −80, CpG +97, and CpG +102) was identified to be methylated by using BGS. All above ten factors were included in a multivariate Cox proportional hazards model to adjust for the effects of covariates. With the multivariate analysis, the methylation of combined CpG (−116, −80, +97, and +102) sites (HR =1.752, P = 0.010) was identified as the independent predictor with the OS of gastric cancer patients postoperatively, as was the N stage (HR =1.452, P <.001) (Table 1). In addition, we demonstrated that methylation of combined CpG (−106, −70, +100, and +111) sites had the smaller AIC value than N stage (57.586 v 72.070), representing optimum prognostic predictor of gastric cancer.

### Correlation Analysis

The results of correlation analysis between the methylation of RNF180 promoter and patient demographics are shown in Table 2. Patients with eight or more methylated CpG sites had significantly higher extragastric lymph node metastatic rate (49.76% v 34.87%; P = 0.010) and methylated rate of combined CpG (−116, −80, +97, and +102) sites (91.70% v 13.33%; P < 0.010) than those with seven or less methylated CpG sites. Patients with the CpG −80 methylation had significantly higher N3 stage lymph node metastatic rate (46.09% v 35.66%; P = 0.024).

**Table 2:** Correlation analysis between methylation of RNF180 and clinicopathological characteristics

Characteristics	T stage				N stage				Location of lymph node metastasis			Lauren classification		
	T1	T2	T3	T4	N0	N1	N2	N3	Intestinal	Diffuse	Mixed	No	Perig- -astric	Extragastric
Methylated CpG site count of RNF 180 promoter														
Seven or less	2	8	10	175	53	28	49	65	53	74	68	56	133	6
Eight or more	2	9	16	178	45	31	38	91	45	58	102	56	140	9
P value	0.748				0.099				0.010			0.767		
Methylated status of combination CpG (−116, −80, +97, and +102) sites														
U	2	10	10	164	48	25	49	64	48	69	69	54	126	6
M	2	7	16	189	50	34	38	92	50	63	101	58	147	9
P value	0.630				0.117				0.111			0.819		
Methylated status of CpG −116														
U	2	10	12	206	58	31	56	85	58	78	94	61	162	7
M	2	7	14	147	40	28	31	71	40	54	76	51	111	8
P value	0.664				0.397				0.745			0.467		
Methylated status of CpG −80														
U	2	16	14	240	68	37	70	97	68	94	110	80	181	11
M	2	1	12	113	30	22	17	59	30	38	60	32	92	4
P value	0.060				0.024				0.458			0.559		
Methylated status of CpG +97														
U	2	12	14	211	61	32	59	87	61	86	92	69	163	7
M	2	5	12	142	37	27	28	69	37	46	78	43	110	8
P value	0.714				0.223				0.129			0.541		
Methylated status of CpG +102														
U	2	14	17	219	62	32	62	96	62	88	102	70	170	11
M	2	3	9	134	36	27	25	60	36	44	68	42	102	4
P value	0.358				0.200				0.491			0.700		

Abbreviations: U, unmethylation; M, methylation.

## DISCUSSION

Despite the decrease in incidence of gastric cancer in recent decades, the disease remains the second leading causes of cancer death worldwide. Gastric cancer continues to be a worldwide health problem, with a frequency that varies greatly across different geographic locations. Gastric cancer is a relatively frequent neoplasm in Asia, yet contributes substantially to the burden of cancer deaths [7]. In Asia, age-adjusted incidences of gastric cancer are up to 10 times that in the USA. The highest rates occur in Japan and Korea, with 42% of worldwide cases occurring in China [8]. Currently, although recently patients appear to benefit from surgery, perioperative chemotherapy, postoperative chemoradiotherapy, and postoperative chemotherapy, the overall prognosis for advanced disease remains poor [9]. The overall 5-year survival rate of patients with resectable gastric cancer ranges from 10% to 30% [10]. Most patients present with advanced pathologic stage and can expect a median survival of 24 months in tumors resected with curative intent. Accurately diagnostic staging and precisely prognostic prediction are critical for improvement the overall patient outcomes and the individualized therapies. It is consensus that the latest edition TNM classification is the best stage of gastric cancer for clinical treatment and prognostic evaluation. However, the limitation of the current stage of gastric cancer indicates that elements based on molecular or immunohistochemical features of the tumor are promising to be practical for the majority of gastric cancers for the near future [11]. So far, relatively low sensitivity and specificity in the diagnosis and prognosis of gastric cancer limits the further use of the commonly used biomarkers of gastric cancer [12]. Therefore, searching for the highly efficient markers to gastric cancer is urgently required for establishment.

Protein degradation by the proteasomes plays a vital role in controlling the level of proteins involved in diverse cellular processes, including differentiation, proliferation and apoptosis. The ubiquitin proteasome system (UPS) is an essential metabolic constituent of cellular physiology that tightly regulates cellular protein concentrations with specificity and precision to optimize cellular function. In the ubiquitin proteasome pathway, substrates are marked by covalent linkage to ubiquitin for degradation [13]. Ubiquitination involves highly specific enzyme cascades such as E1 ubiquitin-activating enzyme, E2 ubiquitin-conjugating enzyme and E3 ubiquitin-protein ligase. RNF180 is a member of E3 ubiquitin ligases which plays a key role in the UPS function by determining the specificity and timing of ubiquitination and subsequent degradation of its substrates [14]. Therefore, RNF180 perhaps participate in a variety of biological and pathological processes in theory. In the previous study, RNF180 transcript was identified to be specially silenced or down-regulated in gastric cancer cells and primary gastric cancer tissues, and the promoter methylation was found to directly mediate RNF180 transcription silencing which significantly alter the malignant biological characteristics of gastric cancer cell (growth, and apoptosis) [6]. Further, RNF180 hypermethylation was detected in the 76% of gastric cancer tissues, but not in normal controls, indicating that RNF180 methylation is a common event in gastric cancers [6]. Lastly, loss or down-regulation of RNF180 was also identified to be associated with a significantly increased risk of cancer-related death of 149 gastric cancer patients [6]. In this study, we detected the differences of RNF180 expression in gastric cancer and paired adjacent non-tumor tissues with protein and mRNA detection methods. With the immunohistochemical staining and Western Blot detection, we detected that RNF180 protein expression in gastric cancer tissues was significantly lower than that in paired adjacent non-tumor tissues. In addition, the mRNA expressive level of RNF180 was also demonstrated to be much lower in gastric cancer tissues than that in paired adjacent non-tumor tissues. Therefore, we thought that the abnormal expression of RNF180 in gastric cancer was associated with the aberrant mRNA transcription and protein translation events, which might be resulted from the DNA promoter methylation as the previous elucidation [6].

It is a consensus that DNA promoter methylation of tumor suppressor genes should be considered to be involved in human carcinogenesis. RNF180 methylation was so frequently detected in gastric cancer tissue indicting that RNF180 is likely a tumor suppressor in the previous study [6], we decided to initially detect the methylation of RNF180 promoter with the qualitative analysis. With the MSP analysis, we found 67.16% (45/67) gastric cancer tissues presented with RNF180 promoter methylation and none of 25 normal gastric mucosal tissues presented with RNF180 promoter methylation. Of these 45 tissues, 33 cases (73.33%) are partial methylation of RNF180 promoter, which indicates the various methylated levels of RNF180 promoter were potentially associated with gastric canceration. In view of the value of prognostic prediction of the methylation RNF180 promoter in gastric cancer reported previously [6], we decided to quantitatively detecte the methylated levels of RNF180 promoter in the large scale gastric cancer tissues to evaluate its applicability as the important prognostic predictor of patients.

Unlike the previous study, we adopted the BGS method with no less than five clones of each gastric cancer sample for enhancement the detection accuracy of the methylation of CpG sites of RNF180 promoter in this study. With this method, we found that both methylated CpG site count and location of methylated CpG sites of RNF180 promoter were negatively associated with the OS of 400 gastric cancer patients. Although four hypermethylated CpG sites (CpG −116, CpG −80, CpG +97, and CpG +102) were significantly associated with the poor survival of all gastric cancer patients respectively, the multivariate survival analysis demonstrated that the methylation of combined CpG (−116, −80, +97, and +102) sites was independent predictor of prognosis rather than any methylation of CpG site alone. In addition, we demonstrated that the methylation of combined CpG (−116, −80, +97, and +102) sites had smaller AIC value than N stage, which indicated that the methylation of combined CpG (−116, −80, +97, and +102) sites was more superior for precisely prognostic evaluation. With the correlation analysis, we found that almost patients (91.7%) with eight or more methylated CpG sites which indicated the poorer survival had the methylation of combined CpG (−116, −80, +97, and +102) sites. Therefore, we deduced that the methylation of the above-mentioned four hypermethylated CpG sites (CpG −116, CpG −80, CpG +97, and CpG +102) should be deemed as the potentially key sites which were used for the methylated detection to predict the prognosis of gastric cancer patients in clinic. However, we could not exclude completely the congenerous effects of other CpG sites of RNF180 promoter contributing to the progression of gastric cancer owing to no methylated CpG site in normal gastric mucosal tissues.

Another important finding of this study is the significant correlation between the methylated CpG site of RNF180 promoter and lymph node metastasis. Ni et al [15] reported that high activity ubiquitin-proteasome pathway in both patient samples and the BxPC-3 pancreatic cancer cell line was detected, and the status of ubiquitinated gelsolin is related to lymph node metastasis of pancreatic cancer. Wu et al [16] found the abnormal activation of ubiquitin-proteasome pathway accelerated the degradation of I kappa B alpha to increase NF-kappa B expression in gastric carcinoma tissues, which was significantly associated with the increase of lymph node metastasis. Therefore, we thought that RNF180, as a member of E3 ubiquitin ligases, might take part in above molecular events to promote the lymph node metastasis from gastric cancer. Extragastric lymph node metastasis from gastric cancer is the absolutely important predictor of disease relapse and poor survival [17]. Approximately half of patients with eight or more methylated CpG sites of RNF180 promoter presented with the extragastric lymph node metastasis, which was an important reason for explanation the poor survival of these patients. CpG −80 methylation of RNF180 promoter was identified to be significantly associated the N3 stage lymph node metastasis from gastric cancer in this study, which was a novel clue for the further mechanism research of the biological effects of each CpG site contribution to metastasis of gastric cancer.

There are some limitations to our study. All samples are obtained from Chinese population in this study, which perhaps result in little bias of detection results comparing to the other race. In viewing of about 42% of worldwide gastric cancer patients occurring in China, the large scale patient-based samples are capable of possessing the certain of representative significance. Besides, this is first report of the CpG site methylation of RNF180 promoter to evaluate the prognosis of gastric cancer. We found that only few methylated CpG sites of RNF180 promoter was appropriate to predict the survival of gastric cancer. Future research should focus on the effects of the given CpG sites contribution to the biological behaviors of gastric cancer cells and the targeted therapy to the given CpG sites of RNF180 promoter.

## PATIENTS AND METHODS

### Data Source

After approval from the Tianjin Medical University Cancer Hospital institutional review board, data from the cancer registry of the Tianjin Cancer Institute was obtained. Oral and written inform consents were obtained from the patients who were included in this study. Information which was obtained through participating cancer registry included: age, gender, tumor location, tumor size, depth of tumor invasion (T stage, according to the seventh edition UICC TNM classification for gastric cancer), number of metastatic lymph nodes (N stage, according to the seventh edition UICC TNM classification for gastric cancer), extent of lymph node metastasis, Lauren classification, and follow-up vital status.

### Patients and Study Samples

For RNF180 promoter methylation analysis, we collected 400 fresh tumor tissues from gastric cancer patients who underwent curative gastrectomy between April 2003 and December 2007 at the Department of Gastroenterology, Tianjin Medical University Cancer Hospital. In addition, a cohort of 25 normal gastric mucosal epithelial tissues derived from normal people between 2004 and 2007 at the Department of Endoscopic Examination and Treatment, Tianjin Medical University Cancer Hospital. All the tumor and normal gastric mucosal epithelial tissues were histologically verified. The patients were not administered radiation, chemical or biological treatment prior to surgery. The clinicopathological characteristics of these 400 gastric cancer patients are summarized in Table 1. The patients' consent was obtained for the use of the tissue samples and records, and the study protocol was approved and permission for use of the clinical data was given by the Institutional Research Ethics Committee of Tianjin Medical University Cancer Hospital.

### Surgical Treatment

Curative resection was defined as a complete lack of grossly visible tumor tissue and metastatic lymph nodes remaining after resection, with pathologically negative resection margins. Primary tumors were resected en bloc with limited or extended lymphadenectomy (D1 or D2-3 according to the Japanese Gastric Cancer Association (JGCA)). Surgical specimens were evaluated as recommended by the seventh UICC TNM classification for gastric cancer.

### Immunohistochemistry

67 of 400 gastric cancer tissues and 67 paired adjacent non-tumor tissues were detected the RNF180 expression by using the immunohistochemical detection for demonstration the difference of RNF180 protein expression between two groups of tissues. Paraffin sections (4μm thick) were deparaffinized and rehydrated. Antigen retrieval treatment was done at 95°C for 40 minutes in 0.01 mol/L sodium citrate buffer (pH 6.0), and endogenous peroxidases were blocked using 3% hydrogen peroxide for 30 minutes. Purchased antibody was goat anti- RNF180 (Santa, sc-137731X, 1:200 dilution). All sections were incubated overnight with the primary antibody at 4°C. The sections were then treated with peroxidase using the labeled polymer method with Zhongshan Peroxidase (Beijing, China) for 30 minutes. Antibody binding was visualized using the Avidin Biotin Complex (ABC) Elite Kit and 3,3'-diaminobenzine according to the manufacturer's instructions (City Key Laboratory of Tianjin Cancer Center, China). Sections were then counterstained in hematoxylin. For general negative controls, the primary antibody was replaced with PBS.

### Microscopic Assessment of RNF180 Protein Expression

All sections were assessed blindly by two independent observers, and in cases of assessing disagreement a third independent assessment was performed. Staining for RNF180 protein was considered potentially positive if there was cytoplasmic staining. The grade of staining intensity of RNF180 protein was rated on a scale from “-” to “+++”, with “-”, indicating no staining; “+”, weak staining; “++”, moderate staining; and “+++”, strong staining. The intensity scores of “++” and “+++” were considered positive staining [18].

### Western Blotting Analysis

67 of 400 gastric cancer tissues and 67 paired adjacent non-tumor tissues were detected the RNF180 expression by using the Western Blotting detection for demonstration the difference of RNF180 protein expression between two groups of tissues. All tissue specimens were respectively added to 1 mL of 100 mmol/L Tris/HCl (pH 7.5), 100 mmol/L NaCl, 0.5% sodium deoxycholate, 1 mmol/L ethylenediaminetetraacetic acid, 1% Nonidet P-40, 0.1% sodium dodecyl sulfate, and protease inhibitor. After blocking, 50 ug sample was incubated for 60 minutes with a goat anti- RNF180 (Santa, sc-137731X, 1:1000 dilution) at room temperature. Gel Imager system (Asia Xingtai Mechanical and Electrical Equipment Company, Beijing, China) to analyze images and to determine gray values.

### Semi-quantitative Reverse Transcription Polymerase Chain Reaction (RT-PCR) Analysis

67 of 400 gastric cancer tissues and 67 paired adjacent non-tumor tissues were detected the RNF180 expression by using the Semi-quantitative Reverse Transcription Polymerase Chain Reaction (RT-PCR) detection for demonstration the difference of RNF180 mRNA expression between two groups of tissues. For the RNF180 semi-quantitative RT-PCR, RNA was extracted from gastric adenocarcinoma tissue, and adjacent non-tumor tissues using Trizol reagent (Invitrogen, Carlsbad, CA) according to the manufacturer's instructions. Total RNA was reverse transcribed to cDNA in a 20 ul volume using Reverse Transcription kit (Invitrogen, Carlsbad, CA). Primers designed and utilized for RNF180 was as follows: Forward sequence: 5'-TCTGACTTTCCTGATGGACC TG-3', and Reverse sequence: 5'-CCTGAG TATTTACCCTGCTTCTGT-3'. The GAPDH gene was used as an endogenous control for quantitative DNA-PCR. Primers designed and utilized for GAPDH was as follows: Forward sequence: 5'-GAAGGTGAAGGTCGGAGTC-3', and Reverse sequence: 5'-GAAGATGGT GATGGGATTTC-3'. The PCR Cycling conditions for all sequences were 35 cycles of denaturation at 95 °C for 3 minutes, annealing at 94 °C for 30 seconds, and extension at 56°C for 30 seconds followed by a final extension at 72°C for 8 minutes. All PCR product electrophoreses were performed on a 2% agarose gel with ethidium bromide and visualized using the Gel Imager system (Asia Xingtai Mechanical and Electrical Equipment Company, Beijing, China).

### DNA extraction and Sodium bisulfite treatment

Genomic DNA was extracted from gastric cancer tissues and normal gastric mucosal epithelial tissues using QIAamp DNA mini kit (Qiagen, Valencia, CA) following the manufacturer's instructions. Sodium bisulphite modification of genomic DNA was performed by using the EZ DNA Methylation-GoldTM Kit (Zymo Research, Hornby, Canada).

### Methylation-specific PCR (MSP)

67 of 400 gastric cancer tissues and 25 normal gastric mucosal tissues were detected the qualitatively methylated analysis of RNF180 promoter with the methylation-specific PCR (MSP). RNF180 primers detecting methylated (M) or unmethylated (U) alleles of the RNF180 promoter were: RNF180-MF, 5'-TTTGCGCGGGGTTAAAGTTC-3' and RNF180-MR, 5'-CGATACCGATT CGACGAAACG-3' for methylated alleles; RNF180-UF, 5'-TGTTTGTTTGT GTGGGGTTAAAGTTT-3' and RNF180-UR, 5'-CAACAACAATACCAATTC AACAAAACA-3' for unmethylated alleles. MSP was performed for 25 cycles using Ampli Taq-Gold (methylation-specific primer, annealing temperature 600°C; unmethylation specific primer, annealing temperature 580°C). MSP primers were first checked for not amplifyling any unbisulfited DNA and the specificity of MSP was further confirmed by direct sequencing of some PCR products. PCR reactions were resolved on a 2% agarose gel.

### Bisulphite Genomic Sequencing (BGS)

All 400 gastric cancer tissues and 25 normal gastric mucosal tissues were detected the quantitatively methylated analysis of RNF180 promoter with the bisulphite genomic sequencing (BGS). Hot start PCR with the bisulfite-treated DNA was performed with a 318bp PCR product spanning promoter region −192bp to 126bp relative to the transcription start site of RNF180. 43 CpG sites were contained in the promoter region of RNF180. The sequences of PCR primers were as follows: F: 5'-GTGGTTTTGGTA AGGGGATGAT-3'; R: 5'-CCAACAACCAAACTCTAAAAACTC-3'. The purified PCR products were cloned into the pUC18-T vector (Biodee, Beijing, China), and no less than five clones for each sample were randomly selected and sequenced by Shanghai Sangon Co.(Shanghai, China).

### Follow-Up

After curative surgery, all patients were followed every 3 or 6 months for 2 year at outpatient department, every year from the third to fifth years, and then annually thereafter until the patient died. The median follow-up for the entire cohort was 41 months (range: 1-104). The follow-up of all patients who were included in this study was completed in December 2012. Ultrasonography, CT scans, chest X-ray, and endoscopy were obtained with every visit.

### Statistical Analysis

The median OS was determined by using the Kaplan-Meier method, and log-rank test was used to determine significance. Factors that were deemed of potential importance on univariate analyses (P <0.05) were included in the multivariate analyses. Multivariate analysis of OS was performed by means of the Cox proportional hazards model. Hazard ratios (HR) and 95% CI were generated. The Bayesian information criterion (AIC) values within a Cox proportional hazard regression model was calculated for different categors to measure theirs discriminatory ability. A smaller AIC value indicated a better model for predicting outcome [19]. With the cut-point survival analysis [20], the optimal cutoff for CpG site conut was identified to be seven. Significance was defined as P < 0.05. All statistical analyses were performed with SPSS 18.0 software.
